# Physiotherapy With Telerehabilitation in Patients With Complicated Postoperative Recovery After Esophageal Cancer Surgery: Feasibility Study

**DOI:** 10.2196/16056

**Published:** 2020-06-09

**Authors:** Maarten A van Egmond, Raoul H H Engelbert, Jean H G Klinkenbijl, Mark I van Berge Henegouwen, Marike van der Schaaf

**Affiliations:** 1 Department of Rehabiltation Amsterdam University Medical Centers University of Amsterdam Amsterdam Netherlands; 2 Faculty of Health Center of Expertise Urban Vitality Amsterdam University of Applied Sciences Amsterdam Netherlands; 3 Faculty of Health European School of Physiotherapy Amsterdam University of Applied Sciences Amsterdam Netherlands; 4 Department of Surgery Gelre Hospital Apeldoorn-Zutphen Apeldoorn Netherlands; 5 University of Amsterdam Amsterdam Netherlands; 6 Department of Surgery, Cancer Center Amsterdam Amsterdam University Medical Centers University of Amsterdam Amsterdam Netherlands

**Keywords:** physical therapy modalities, telerehabilitation, telemedicine, esophageal neoplasms, surgical oncology

## Abstract

**Background:**

Improvement of functional status with physiotherapy is an important goal for patients with postoperative complications and an increased length of hospital stay (LoS) after esophagectomy. Supervised physiotherapy with telerehabilitation instead of conventional face-to-face care could be an alternative to treat these patients in their home environment after hospital discharge (T0), but its feasibility has not yet been investigated in detail.

**Objective:**

The aim of this study was to investigate the feasibility of a 12-week supervised postoperative physiotherapy intervention with telerehabilitation for patients with esophageal cancer who underwent esophagectomy and had postoperative complications or who had an increased LoS. The secondary objective was to investigate the preliminary effectiveness of telerehabilitation on functional recovery compared with usual care.

**Methods:**

A prospective feasibility study with a matched historical comparison group was performed. Feasibility outcomes included willingness and adherence to participate, refusal rate, treatment duration, occurrence of adverse events, and patient satisfaction. Secondary outcome measures were measurements of musculoskeletal and cardiovascular functions and activities according to the domains of the International Classification of Functioning, Disability and Health.

**Results:**

A total of 22 patients with esophageal cancer who underwent esophagectomy and had postoperative complications or an increased LoS were included. The mean age at surgery was 64.55 (SD 6.72) years, and 77% (17/22) of patients were male. Moreover, 15 patients completed the intervention. Patient adherence was 99.8% in the first 6 weeks and dropped to 75.6% in the following 6 weeks, with a mean difference of −24.3% (95% CI 1.3 to 47.2; *P*=.04). At 3 months post operation, no differences in functional status were found between the intervention group and the matched historical comparison group.

**Conclusions:**

This study showed that a postoperative physiotherapeutic intervention with telerehabilitation is feasible for patients with postoperative complications or an increased LoS after esophageal cancer surgery up to 6 weeks after T0.

## Introduction

### Background

Surgical resection of the esophagus is the primary curative treatment for patients with esophageal cancer and is associated with a high risk of postoperative complications, varying from 25% to 60% [1,2]. This leads to an increased length of hospital stay (LoS) and a delayed postoperative recovery, with a significant decline in physical function in the first 3 months after surgery [2,3].

It has been demonstrated in many surgical populations that improving preoperative functional status by exercise training had a positive effect on long-term postoperative outcomes [4,5]. However, recent studies have shown that preoperative functional status was not associated with postoperative complications in patients treated with esophagectomy, justifying the need to focus on treating these patients in the postoperative phase [6,7].

Patients with postoperative complications after esophagectomy often have fatigue, decreased exercise capacity, and disability such as impaired walking capacity and their recovery could take up to one year and beyond [3,8]. These symptoms are explained by altered cardiopulmonary function, generalized muscle weakness, and malnutrition, and physiotherapists play an important role in improving these aspects of physical functioning [9].

### Telerehabilitation as an Alternative to Face-to-Face Care

Instead of face-to-face care, postoperative physiotherapy can also be streamed by telerehabilitation. Telerehabilitation is a medium to provide physiotherapy with electronic health (eHealth), defined as *the delivery of rehabilitation services to patients at a distance using information and communication technologies* [10]. Telerehabilitation has shown to be a valuable tool in improving postoperative outcomes and functional recovery in surgical patients, where patients considered reduced barriers for travel, flexible exercise hours, and the ability to directly integrate exercises into daily life as positive [11,12].

Moreover, telerehabilitation interventions have been valuable to overcome discontinuities that may arise in communication between hospital and primary care, where physiotherapists may have a lack of knowledge about how to treat patients after a highly complex surgery [13].

There is evidence showing positive effects of physiotherapy with telerehabilitation on clinical outcomes in patients with cancer, patients with cardiac disease, and patients with musculoskeletal disorders, but information on the feasibility of this intervention in the postoperative phase of patients with esophageal cancer treated with esophagectomy is lacking [14,15].

### Objectives

Therefore, the primary objective of this prospective feasibility study was to investigate the feasibility of a 12-week supervised postoperative telerehabilitation program for patients with esophageal cancer who underwent esophagectomy and had postoperative complications or who had an increased LoS. The secondary objective was to investigate the preliminary effectiveness of telerehabilitation on functional recovery compared with a matched historical comparison group receiving usual care.

## Methods

### Ethical Approval

The Medical Ethical Committee (METC) of the Amsterdam University Medical Centers provided ethical approval for this study (NL58388.018.16). All patients provided written informed consent. As this was a feasibility study, sample size calculations have not been performed, and the initial sample size of 30 participants was pragmatically chosen. Patients could leave the study at any time for any reason if they wished to do so without any consequences.

### Study Design

A prospective feasibility study was performed in patients treated with esophagectomy. To assess preliminary effectiveness, the patients who underwent the complete treatment were matched with a historical comparison group of patients who underwent esophagectomy and had postoperative complications, receiving usual face-to-face care between March 2012 and October 2014. We decided to match one case to 2 patients from a historical comparison group to optimize statistical power. Data collected from this historical comparison group were part of a previous study performed by the same research group, from which the METC waived the need for informed consent [6]. Patients were matched for gender, age, American Society of Anesthesiologists Physical Status Classification, comorbidities, Body Mass Index, pulmonary function, surgical procedure, and severity of postoperative complications.

### Participants

Patients were recruited from the surgical wards at the Gastrointestinal Oncologic Centre Amsterdam of the Amsterdam University Medical Centre, located in the Academic Medical Centre, just before discharge from the hospital by the supervising physiotherapist or the investigator. Patients who refused to participate were referred to face-to-face physiotherapy in primary care.

### Inclusion Criteria

Participants were included if they were aged 18 years or older and the primary reason of hospital stay was status after esophagectomy, they had internet access at home, and they signed the informed consent form. Moreover, participants were included if they had a postoperative complication, grade 3a to 4 according to the Clavien-Dindo classification. This 5-scale classification reports surgical complications based on the type of therapy required to treat the complication [16]. Participants were also included if the postoperative LoS was longer than 9 days because they were physically too weak to be discharged earlier. There was an indication for face-to-face physiotherapy in primary care if a patient was not yet able to walk or transfer independently because of loss of muscle strength, mobility, or balance at discharge.

### Exclusion Criteria

Patients were excluded if they were unable to complete self-reported questionnaires, insufficiently able to read or speak Dutch, had cognitive disorders, or had any other severe medical conditions that prevented them from doing unsupervised exercises at home.

### Intervention

Participants received a 12-week supervised home-based telerehabilitation intervention after hospital discharge (T0) in their home environment. Before T0, a physiotherapist from the surgical ward instructed the patient on the telerehabilitation intervention.

**Figure 1:**
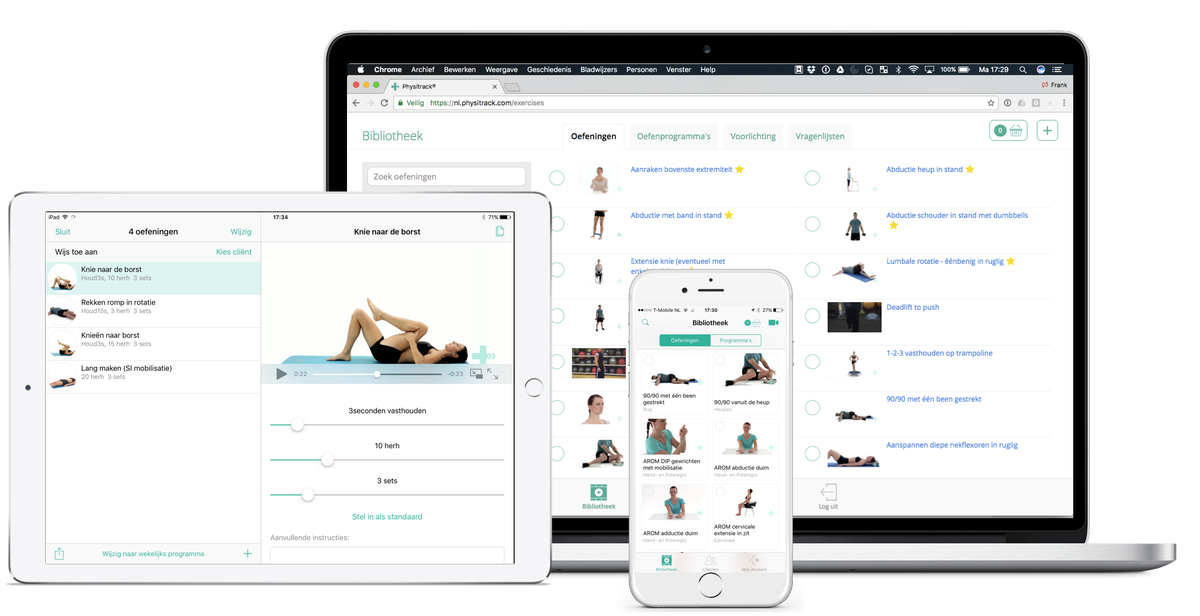
Goal-oriented exercise program created by the physiotherapist, accessible by tablet, mobile phone, or computer (Used with permission from Physitrack).

The telerehabilitation intervention was provided with Physitrack (Physitrack Limited). Physitrack is an eHealth platform that enables physiotherapists to design home exercise programs and track patient adherence. Patients were provided with a goal-oriented exercise program created by the physiotherapist that could be accessed by a tablet, mobile phone, or computer (Figure 1). The physiotherapist accurately monitored the progress of the patient in weekly telephone, email, or video sessions, and exercises were adapted via the eHealth platform if needed. Physitrack had provided their services for this research project free of charge, and they will use the outcomes of this study to improve their services. They were not involved in the design, execution, analysis, and conclusions of this research. Physitrack will only have access to the published paper with its results, with no access to raw data.

The postoperative physiotherapeutic intervention with telerehabilitation was aimed at improving functional status. The intervention lasted 12 weeks with at least two sessions per week, depending on whether the treatment goals were achieved. The exercises were tailored to the patients’ specific condition and needs, which were determined a day before T0. The physiotherapy goals were determined by using the patient-specific complaint list [17]. The exercises were aimed at improving the functional activity level of the patient, by increasing muscle strength, coordination, range of joint motion, and stamina. The intensity and frequency of the functional exercises were determined according to the guidelines of the American College of Sports Medicine [18]. Cardiorespiratory exercises to improve stamina were performed on a moderate-to-vigorous intensity level, measured using the Borg rating of perceived exertion scale (scores 6-20), for at least two sessions per week. Rating of perceived exertion with the Borg scale is a generally used and reliable scale to monitor and evaluate exercise intensity. A score from 13 to 16 relates to the moderate-to-vigorous intensity level, and this allowed us to monitor and adapt the appropriate intensity [19]. Exercises to improve muscle strength were performed 2 to 3 days per week on 60% to 70% of the 1 repetition maximum (moderate-to-hard intensity). We used the Holten curve that relates the percentage of the 1 repetition maximum to the estimated repetitions of that intensity. This allowed us to adapt the exercises without using fitness equipment to measure the 1 repetition maximum directly [18,20].

### Feasibility Outcome Measures

Feasibility outcome measures were calculated for the 15 patients who completed the 12-week supervised home-based telerehabilitation intervention. Feasibility outcomes included refusal rate; adherence to the telerehabilitation intervention operationalized in the amount and duration of email, phone, and video calls conducted by patients and physiotherapists; treatment duration per session; adverse events; and patient satisfaction. Patient satisfaction was recorded with a modified telemedicine satisfaction and usefulness questionnaire (TSUQ), a 30-item Likert-type questionnaire including 3 subscales (usefulness, communication, and user friendliness) at 6 weeks post operation (T1) and at 3 months post operation (T2) [21]. Scores range from 30 to 150, with high scores indicating a higher satisfaction.

The telerehabilitation intervention was considered as feasible if at least an 80% adherence rate was achieved, if no adverse events took place, and if the average total patient satisfaction was higher than 75% (score >120).

### Effectiveness Outcome Measures

Secondary outcome measures on preliminary effectiveness were musculoskeletal and cardiovascular functioning and level of activities according to the domains of the International Classification of Functioning, Disability and Health [22].

Handgrip strength was measured using the Jamar grip strength dynamometer (Lafayette Instrument Company) as a measure of generalized muscle strength [23,24]. Maximal inspiratory pressure was measured as an indicator of inspiratory muscle strength, with a Micro Respiratory Pressure Meter [2,4]. Functional lower extremity muscle function was measured with the 30-second chair stand test (30CST). This test measures extremity strength in relation to demanding functional daily activities such as stair climbing and getting out of a chair [25]. Walking capacity was measured using the 2-min walk test (2MWT) [26].

Fatigue was measured using the Multidimensional Fatigue Inventory [27].

Self-reported activities were measured using the Longitudinal Ageing Study Amsterdam Physical Activity Questionnaire (LAPAQ) in which patients reported the type, frequency, and duration of daily activities in the past 14 days. Health-related quality of life (HRQL) was measured using the European Organization for Research and Treatment of Cancer Quality of Life Questionnaire C30, version 3.0 [28].

The effectiveness of outcome measures was recorded before the start of the intervention (T0) and at T1 and T2.

Standardized operating procedures of all measurements were used to guarantee uniformity and accuracy in operationalization. Trained and experienced physiotherapists performed the standardized measurements.

### Statistical Analysis

Data were analyzed in the Statistical Package for the Social Sciences (version 25.0; IBM SPSS Statistics for Windows, IBM Corp). Statistical tests were analyzed two sided and considered significant with an alpha value ≤.05.

Baseline characteristics were summarized with descriptive statistics, where discrete variables were expressed as counts with percentages, ordinal variables as median and interquartile ranges (P25-75), and continuous variables as mean and standard deviation, and in case of a skewed distribution, they were expressed as median and interquartile range. Differences in outcomes before and after the intervention were determined by using a paired samples *t* test or a Wilcoxon matched-pairs signed rank test for skewed data. Differences between the intervention group and the historical comparison group were tested using a linear mixed model analysis to account for the dependency between observations.

## Results

### Baseline Characteristics

From January 2017 to October 2018, 22 patients with esophageal cancer who underwent esophagectomy were included in the study after obtaining informed consent. The study was terminated after the inclusion of the 22nd patient because we reached a point in data collection after which no new or relevant information emerged with respect to answering the primary research question.

The mean age at surgery was 64.6 (SD 6.7) years, and 77% (17/22) of patients were male. All patients received neoadjuvant chemoradiation therapy. At enrollment, mean pulmonary function expressed as a percent score of the predicted pulmonary function value was 116.1 (SD 18.7) for forced vital capacity, 109.0 (SD 19.3) for forced expiratory volume in 1 second, and 109.2 (SD 30.1) for inspiratory vital capacity. Except for 2 patients, all other patients were surgically treated with a minimally invasive transthoracic esophagectomy. In addition, 36% (8/22) of patients had a hospital stay of more than 9 days. Moreover, 91% (20/22) of patients had postoperative complications, of which 70% (14/22) required a surgical, an endoscopic, or a radiological intervention. Patient characteristics are presented in Table 1.

**Table 1:** Characteristics of the entire study population.

Patient characteristics			Study population (N=22)
Gender (male), n (%)			17 (77)
**ASA^a^ classification, n (%)**			
	I^b^	5 (22)	
	II^c^	10 (45)	
	III^d^	7 (31)	
Age (years), mean (SD)			64.6 (6.7)
BMI^e^, mean (SD)			26.5 (4.4)
**Comorbidities, n (%)**			
	Cardiovascular	7 (31)	
	COPD^f^	0 (0)	
	DM II^g^	2 (9)	
	Cigarette smoking	2 (9)	
**Pulmonary function (percent predicted), mean (SD)**			
	FVC^h^	116.1 (18.7)	
	FEV_1_^i^	109.0 (19.3)	
	IVC^j^	109.2 (30.1)	
**Surgical procedure, n (%)**			
	Transhiatal open	0 (0)	
	Transhiatal minimally invasive	1 (5)	
	Transthoracal open	0 (0)	
	Transthoracal minimally invasive	20 (91)	
	Esophageal resection with colon interposition	1 (5)	
**Clavien-Dindo postoperative complications, n (%)**			
	No complications	2 (9)	
	Grade 1	2 (9)	
	Grade 2	4 (18)	
	Grade 3a	3 (14)	
	Grade 3b	4 (18)	
	Grade 4a	7 (32)	
	Grade 4b	0 (0)	
	Grade 5	0 (0)	

^a^ASA: American Society of Anesthesiologists.

^b^I: healthy person.

^c^II: mild systemic disease.

^d^III: severe systemic disease.

^e^BMI: body mass index is calculated as weight in kilograms divided by height in meters squared.

^f^COPD: chronic obstructive pulmonary disease.

^g^DM II: diabetes mellitus type 2.

^h^FVC: functional vital capacity.

^i^FEV1: forced expiratory volume in the first second of expiration.

^j^IVC: inspiratory vital capacity.

### Feasibility

Of 22 patients, a total of 15 (68%) patients completed the 12-week program. Of the 7 patients who did not complete the study, 1 was discharged to a nursing home after inclusion, 2 quit the study intervention after 3 and 4 weeks because they preferred face-to-face physiotherapy, and 4 patients were withdrawn by the investigator because postoperative treatment required a multidisciplinary approach (n=3) or because of the presence of metastases (n=1). These patients did not systematically differ in baseline characteristics from the patients who completed the program.

The average duration of the treatment program was 11.1 (SD 5.2) weeks. Of the 4671 exercises provided to patients, 1337 (28.62%) were aimed at lower extremity muscle strength, 996 (21.32%) were aimed at respiration, and 1150 (24.62%) were aimed at walking.

Patient adherence, operationalized in the performance rate of exercises to the telerehabilitation intervention, was 99.8% in the first 6 weeks and dropped to 75.6% in the following 6 weeks, with a mean difference of −24.3% (95% CI 1.3 to 47.2; *P*=.04). The accomplishment of treatment goals was the main reason reported for being less or not adherent to the program anymore.

The physiotherapist and patients contacted each other 204 times in 243 weeks, with a minimum of 1 and a maximum of 3 times a week for coaching, for regular follow-ups, and for adjusting the treatment program, dependent on the patient’s needs. Of these 204 direct patient contacts, 1 (0.5%) took place with a video connection, 26 (12.7%) with email, 122 (59.8%) with telephone, and 55 (27.0%) with live contact via home visits.

Total average patient satisfaction (range 30-150) measured at T1 was 135.0 (SD 19.5), with subscores on usefulness (range 10-50) being 44.66 (SD 7.4), communication (range 11-55) being 48.3 (SD 8.1), and user friendliness (range 9-45) being 42.8 (SD 3.2). Patients appreciated weekly follow-ups by telephone or email and especially appreciated the flexibility they had to perform the exercises at home. They rated the telerehabilitation app as user friendly, and they did not miss the physical presence of the physiotherapist to follow the exercise program. No adverse events took place during measurements or exercise sessions. Total average patient satisfaction at T2 was 139.6 (SD 15.4). Textbox 1 provides a selection of quotes provided by participants more than once about experiences with the program.

**Patient experiences.**

Quotes:

- “It gave a lot of confidence to work at home on my recovery with supervision of a PT” [Mrs S, 70 years]
- “I could do the exercises whenever I wanted, that was very convenient” [Mr W, 54 years]
- “Without this program I would never have been that far” [Mr J, 66 years]
- “I should not have thought about going to the physiotherapist twice a week” [Mrs B, 60 years]
- “By practicing at home, I knew what I was doing it for. That was very motivating” [Mr B, 62 years]
- “I missed incentives in the program” [Mr B, 49 years]
- “I did not miss the physical presence of the physiotherapist, I felt that I could always reach him through the app” [Mrs B, 64 years]
- “Along the way, I found the exercise program less relevant, I could already do my daily activities again” [Mr S, 62 years]

### Effectiveness

A total of 15 patients who completed the telerehabilitation program were matched with 30 patients from a historical comparison group for both pre- and postoperative characteristics (gender, age, preoperative pulmonary function, type of surgery, and postoperative complications classified according to Clavien-Dindo). Table 2 provides details about the matching characteristics.

At T0, patients in the intervention group had significantly lower functional capacity measures compared with reference values than patients in the matched historical comparison group (Table 3).

At 3 months post operation, no differences in functional status measures were found between the intervention group and the matched control group (Table 4).

Within the intervention group, 30CST, 2MWT, fatigue, and HRQL improved significantly between T0 and T1 and between T1 and T2, whereas activities of daily life (ADL) decreased significantly between T0 and T1 and improved again between T1 and T2 (Table 5).

**Table 2:** Patient characteristics of the intervention group matched with a historical comparison group.

Patient characteristics		Intervention (n=15)	Matched controls (n=30)
Gender (male), n (%)		11 (73)	22 (73)
**ASA^a^ classification, n (%)**			
	I^b^	3 (20)	5 (16)
	II^c^	8 (53)	15 (50)
	III^d^	4 (26)	10 (33)
Age (years), mean (SD)		62.8 (6.9)	60.3 (7)
BMI^e^, mean (SD)		26.1 (3.5)	25.2 (4)
**Comorbidities, n (%)**			
	Cardiovascular	6 (40)	5 (16)
	COPD^f^	0 (0)	3 (10)
	DM II^g^	1 (7)	1 (3)
	Cigarette smoking	1 (7)	7 (23)
**Pulmonary function (percent** **predicted),** **mean (SD)**			
	FVC^h^	115.0 (20.1)	116.3 (16.2)
	FEV_1_^i^	105.4 (20.1)	110.2 (20.7)
	IVC^j^	114.1 (21.9)	112.0 (16.7)
**Surgical procedure, n (%)**			
	Transhiatal open	0 (0)	0 (0)
	Transhiatal minimally invasive	0 (0)	2 (7)
	Transthoracal open	0 (0)	1 (3)
	Transthoracal minimally invasive	14 (93)	27 (90)
	Esophageal resection with colon interposition	1 (7)	0 (0)
**Clavien-Dindo postoperative complications, n (%)**			
	No complications	2 (13)	11 (37)
	Grade 1	2 (13)	4 (13)
	Grade 2	2 (13)	7 (23)
	Grade 3a	3 (20)	4 (13)
	Grade 3b	2 (13)	1 (3)
	Grade 4a	4 (27)	2 (7)
	Grade 4b	0 (0)	1 (3)
	Grade 5	0 (0)	0 (0)

^a^ASA: American Society of Anesthesiologists.

^b^I: healthy person.

^c^II: mild systemic disease.

^d^III: severe systemic disease.

^e^BMI: body mass index is calculated as weight in kilograms divided by height in meters squared.

^f^COPD: chronic obstructive pulmonary disease.

^g^DM II: diabetes mellitus type 2.

^h^FVC: functional vital capacity.

^i^FEV1: forced expiratory volume in the first second of expiration.

^j^IVC: inspiratory vital capacity.

**Table 3:** Functional status capacity outcome measures at hospital discharge (T0). Beta values represent the differences in functional status between the historical control group and the intervention group at T0.

Functional status outcome	Intervention	Control	Beta	95% CI	*P* value
RHGS^a^ (percent predicted), mean (SD)	92.4 (19.7)	107.9 (23.2)	−15.5	−31.9 to 0.79	.04^b^
LHGS^c^ (percent predicted), mean (SD)	97.1 (20.8)	106.2 (22.4)	−11.9	−26.6 to 2.9	.11^b^
30CST^d^ (percent predicted), mean (SD)	50.8 (31.6)	89.0 (34.4)	−33.2	−53.8 to −12.7	.003^b^
2MWT^e^ (meters), mean (SD)	117.4 (50.6)	154.4 (32.3)	−22.6	−42.7 to −2.5	.03^b^

^a^RHGS: right-hand grip strength.

^b^*P*≤.05 is considered significant.

^c^LHGS: left-hand grip strength.

^d^30CST: 30-second chair stand test.

^e^2MWT: 2-min walk test.

**Table 4:** Within-group differences between hospital discharge (T0) and 3 months post operation (T2) and between-group differences at T2 in measures of functional status. Within-group differences represent the differences in functional status between T0 and T2. Beta values represent the differences in functional status between the historical control group and the intervention group at T2.

Functional status outcome	Within-group differences (T0-T2)^a,b^					Between-group differences at T2	
	Intervention (n=15)		Historical control (n=30)			Beta	
	Mean (95% CI)	*P* value	Mean (95% CI)	*P* value	Mean (95% CI)		*P* value
LHGS^c^	10.4 (0.1 to 20.8)	.048^d^	−4.1 (−8.7 to 0.5)	.08	0.8 (14.2 to −12.7)		.91
RHGS^e^	12.3 (0.9 to 23.7)	.04^d^	−3.2 (−8.9 to 2.4)	.25	−1.0 (−15.3 to 13.3)		.89
MIP^f,g^	—^h^	—	—	—	13.7 (−14.0 to 41.4)		.32
30CST^i^	69.7 (51.6 to 87.8)	<.001^d^	29.8 (18.7 to 40.9)	<.001^d^	5.9 (−15.3 to 27.0)		.58
2MWT^j^	82.4 (53.4 to 111.3)	.001^d^	41.2 (27.3 to 55.1)	<.001^d^	16.8 (−7.6 to 41.2)		.17
ADL^g,k^	—	—	—	—	−444.3 (−1417.0 to 528.3)		.36
Fatigue^g^	—	—	—	—	−3.6 (−16.0 to 8.8)		.55
HRQL^l^	—	—	—	—	3.5 (−9.0 to 16.11)		.57

^a^T0: hospital discharge.

^b^T2: 3 months post operation.

^c^LHGS: left-hand grip strength.

^d^*P*<.05 is considered significant.

^e^RHGS: right-hand grip strength.

^f^MIP: maximal inspiratory pressure.

^g^These measurements were not performed at T0 and therefore were excluded from this analysis.

^h^Missing data.

^i^30CST: 30-second chair stand test.

^j^2MWT: 2-min walk test.

^k^ADL: activities of daily life.

^l^HRQL: health-related quality of life.

**Table 5:** Mean differences in functional status outcomes between hospital discharge and 6 weeks post operation (T1) and between T1 and 3 months post operation in the intervention group (n=15).

Measurements	Δ^a^T0-T1^b,c^ (95% CI)	*P* value	ΔT1-T2^d^ (95% CI)	*P* value
RHGS^e^	7.4 (−5.1 to 19.8)	.22	5.1 (−1.5 to 11.6)	.12
LHGS^f^	9.6 (−0.6 to 19.8)	.06	1.0 (−5.0 to 6.9)	.74
MIP^g,h^	—^i^	—	9.6 (−1.1 to 20.3)	.07
30CST^j^	53.0 (38.5 to 67.5)	<.001^k^	19.0 (10.2 to 27.9)	.001^k^
2 MWT (m)^l^	51.0 (21.9 to 80.2)	.002^k^	30.3 (15.5 to 445.0)	.001^k^
MFI^m^ fatigue	−10.2 (−16.8 to −3.6)	.007^k^	−16.8 (−24.6 to −9.0)	.001^k^
EORTC QLQ C30^n^, (score)	25.6 (14.6 to 36.5)	<.001^k^	14.6 (6.4 to 22.8)	.002^k^
LAPAQ^o^ (kcal/day)	−514.7 (−866.7 to 160.7)	.008^k^	173.6 (9.5 to 337.7)	.04^k^

^a^Δ: mean difference.

^b^T0: hospital discharge.

^c^T1: 6 weeks post operation.

^d^T2: 3 months post operation.

^e^RHGS: right-hand grip strength.

^f^LHGS: left-hand grip strength.

^g^MIP: maximal inspiratory pressure.

^h^These measurements were not performed at T0 and therefore were excluded from this analysis.

^i^Missing data.

^j^30CST: 30-second chair stand test.

^k^*P*<.05 is considered significant.

^l^2MWT: 2-min walk test.

^m^MFI: Multidimensional Fatigue Inventory; scores range from 20 to 100, with a higher score representing more fatigue and reduced activity/motivation.

^n^EORTC QLQ C30: European Organization for Research and Treatment of Cancer Quality of Life Questionnaire C30; scores range from 0 to 100, with high scores indicating a better quality of life.

^o^LAPAQ: Longitudinal Ageing Study Amsterdam physical activity questionnaire; total amount of activities in kilocalories per day.

## Discussion

### Principal Findings

To our knowledge, this is the first study demonstrating that postoperative physiotherapy with telerehabilitation is feasible in patients with postoperative complications after esophagectomy, primarily in the first 6 weeks after T0. This is in line with a study by Latham et al [11], who stated that telerehabilitation is a valuable tool to manage postoperative outcomes and functional progress directly after T0 in a patient’s home environment.

The adherence rates were significantly higher in the first 6 weeks after T0 than in the following 6 weeks, where patients reported that they were generally more able to perform their ADL and were less dependent on the telerehabilitation intervention, which might explain the lower adherence rates despite a further incline in functional status. From a functional perspective, these lower adherence rates should be interpreted as a desired outcome, because it illustrates the patient’s gradual independence of physiotherapeutic care.

The consistently high patient satisfaction rates of the telerehabilitation intervention in our study are confirmed in a systematic review by Mair et al [29], who stated that the greatest advantages experienced by patients were increased accessibility of specialist expertise, increased flexibility, less travel required, and reduced waiting times. This is also in agreement with the study by Moffet et al [30], who investigated patient satisfaction with in-home telerehabilitation after total knee arthroplasty and found similar results, concluding that patient satisfaction was at least equal to conventional health care delivery.

In this study, we compared patients who underwent the telerehabilitation program with a historical comparison group of patients receiving usual care and found equal functional status outcome measures at T2. This is in line with studies that found telerehabilitation interventions to be equally effective as usual care on at least one outcome measure; however, overall significant evidence in favor of telerehabilitation was still lacking [31,32].

Despite the similar functional outcomes at T2, it has to be noted that most of the functional status outcome measures of our intervention group at T0 were significantly lower than those of the matched historical comparison group. It could be argued that the intervention group gained more progress on functional status because of the physiotherapeutic treatment with telerehabilitation, in comparison with the matched historical comparison group, ultimately resulting in equal outcomes at T2.

Within the intervention group, most of the functional outcome measures significantly improved between T0-T1 and T1-T2, apart from ADL that significantly decreased during the first 6 weeks of the intervention and was restored in the following 6 weeks. A possible explanation could be that after T0, patients mostly stayed at home because they felt too weak to keep up with their ADL. Moreover, in the first 6 weeks, the telerehabilitation intervention primarily focused on increasing muscle strength of the lower extremities. After 6 weeks, the shift was gradually made toward implementing the exercises in daily life, finally resulting in a significant increase in ADL in the following 6 weeks.

### Limitations

This study has intrinsic limitations. First, only 22 patients were included in this study, of which 15 patients completed the study. This might limit the generalizability of our findings. However, despite the small sample size, the included participants represented the population of interest in terms of baseline characteristics and postoperative complications. Moreover, inclusion was terminated after the inclusion of the 22nd participant because no new findings were to be expected with adding new participants to the study.

Second, this study was not a pilot feasibility trial, where patients were randomly assigned either to the intervention group or a control group to determine the effectiveness of investigational treatment. Instead, we compared the intervention group with a matched historical comparison group. Therefore, bias could not be ruled out completely.

We were not able to compare functional status outcome measures half way through the telerehabilitation intervention because the historical controls were not measured at T1.

Third, patient satisfaction was measured with a modified TSUQ that had not been validated in this specific population. Kairy et al [33] in their systematic review investigating clinical outcomes, clinical process, health care utilization, and costs associated with telerehabilitation concluded that patient satisfaction ratings were generally high, irrespective of the population. However, they also stated that operationalization and standardization of satisfaction were frequently lacking and too much focus was on the technology aspect instead of aspects of service delivery. The satisfaction questionnaire we used addressed both aspects, and therefore, we are confident that the satisfaction ratings were representative of the telerehabilitation intervention provided.

### Conclusions

This study shows that patients are able to improve their functional status by doing functional exercises in their own meaningful environment supported by telerehabilitation and tablet use with distant guidance from an experienced physiotherapist. The feasibility of the physiotherapeutic intervention with telerehabilitation for this specific patient category has implications for (re)organizing postoperative physiotherapeutic care in the patient’s home environment. Telerehabilitation cannot replace face-to-face physiotherapy as physical examination remains to be necessary, but taking into account positive adherence rates and satisfaction, we strongly suggest considering this way of treatment delivery for patients with esophageal cancer treated with surgery and having postoperative complications, especially in the first 6 weeks after T0. We also recommend investigating the potential cost-effectiveness of telerehabilitation compared with usual care. Although we found equal functional status outcomes in both the intervention group and the historical comparison group at T2, we suggest performing a randomized controlled trial to draw firm conclusions on its effectiveness.

## Abbreviations

2MWT: 2-min walk test
30CST: 30-second chair stand test
ADL: activities of daily life
eHealth: electronic health
HRQL: health-related quality of life
LAPAQ: Longitudinal Ageing Study Amsterdam Physical Activity Questionnaire
LoS: length of hospital stay
METC: Medical Ethical Committee
TSUQ: telemedicine satisfaction and usefulness questionnaire
